# The Influence of Distance and Lateral Offset of Follow Me Robots on User Perception

**DOI:** 10.3389/frobt.2020.00074

**Published:** 2020-06-03

**Authors:** Felix Wilhelm Siebert, Jacobe Klein, Matthias Rötting, Eileen Roesler

**Affiliations:** Department of Psychology and Ergonomics, Technische Universität Berlin, Berlin, Germany

**Keywords:** human robot interaction, proxemics, human following robots, affinity for technology, robot movement conventions

## Abstract

Robots that are designed to work in close proximity to humans are required to move and act in a way that ensures social acceptance by their users. Hence, a robot's proximal behavior toward a human is a main concern, especially in human-robot interaction that relies on relatively close proximity. This study investigated how the distance and lateral offset of “Follow Me” robots influences how they are perceived by humans. To this end, a Follow Me robot was built and tested in a user study for a number of subjective variables. A total of 18 participants interacted with the robot, with the robot's lateral offset and distance varied in a within-subject design. After each interaction, participants were asked to rate the movement of the robot on the dimensions of comfort, expectancy conformity, human likeness, safety, trust, and unobtrusiveness. Results show that users generally prefer robot following distances in the social space, without a lateral offset. However, we found a main influence of affinity for technology, as those participants with a high affinity for technology preferred closer following distances than participants with low affinity for technology. The results of this study show the importance of user-adaptiveness in human-robot-interaction.

## Introduction

The shift of robotic systems away from structured and standardized settings into unstructured everyday environments is accompanied by the emergence of a number of challenges for the interaction between humans and robotic systems (Sheridan, 2016). Hence, the behavioral design of robots in responsive spatiotemporal collaboration with humans has been moved into the research focus (Honig et al., 2018). Previous research has shown that the successful integration of autonomous service robots into everyday life environments is highly dependent on the human perception of robots' behavior (Kruse et al., 2013). Consequently, there is a need for guidelines which ensure that robots' behavioral design leads to social acceptance and comfort of humans while interacting with robots (Maehara and Fujinami, 2018). As an example, the passing distance of mobile robots during human-robot encounters has been shown to significantly influence human comfort and discomfort (Pacchierotti et al., 2005; Walters et al., 2005a; Nomura et al., 2007; Lauckner et al., 2014). Further, individuals' experience with technology and attitudes toward robots have been shown to influence preferences in robot distance (Walters et al., 2005b; Takayama and Pantofaru, 2009; Mumm and Mutlu, 2011).

A special case of collaborative mobile robots are so called *human-following* robots (or *Follow Me* robots), which follow their users and transport individual property, e.g., after grocery shopping or while doing sports. Potentially, the most basic behavioral design for these types of robots would be to copy the movement of their users while maintaining a predictable following distance, with the expectation that this is perceived as pleasant and comfortable (Honig et al., 2018). Research has shown however, that the nature of Follow Me robots influences users' perception of their following distance, i.e., since the items carried by the robot are often personally relevant, e.g., when carrying a wallet, a close human-robot proximity is preferred over a larger one (Honig et al., 2016). It is hypothesized that the presence of a personal item on the robot changes the human-robot relation (Honig et al., 2016).

The influence of entity-entity relation on preferred and comfortable distance adjustment has been researched for human-human distances in the field of proxemics, in which Hall et al. (1968) hypothesized that the interpersonal environment is divided into an *intimate, personal, social*, and *public* distance. Depending on the relation of humans toward each other, the same distance can be perceived as comfortable or uncomfortable, e.g., the distance to a stranger will be perceived as comfortable if he or she is in the public zone, but not if he or she is in the personal zone. The transfer of Hall's work (Hall et al., 1968) to the field of human-robot interaction is based on the assumption that robots are perceived as social entities (Walters et al., 2005a) to which humans react in a way comparable to humans.

Hence, in this study we apply proxemics, i.e., the subdivision of the space that surrounds humans, to the spacing of Follow Me robots in their collaboration with humans. While there are already a number of studies on the dynamic interaction with robots (Walters et al., 2005a, 2011; Gockley et al., 2007; Morales et al., 2014; Honig et al., 2016) we aim to derive more comprehensive indications for movement conventions in the dynamic interaction with robots by systematically varying two variables in this experiment. Apart from proxemics, which categorize distances, the linear movement in the interaction with Follow Me robots requires the establishment of a convention for the *lateral offset* of the robot in relation to the human user. This lateral offset has been defined as the *following angle* in some research (Honig et al., 2016; Karunarathne et al., 2018). While one study has found a preference for *straight*, or 0° *following* angles when participants are confronted with varying offsets (Honig et al., 2016), in the majority of studies the lateral offset is not varied but fixed (for an overview see Honig et al., 2018), which prohibits a comparison of preferences for lateral offsets in the same research environment. Since humans prefer a lateral offset when walking with other humans (Costa, 2010), a preference for 0° following angles in the interaction with Follow Me robots would indicate a changed lateral preference in human robot interaction. While studies have researched the technical implementation of lateral offset following (Morales et al., 2012, 2014) and straight following (Gockley et al., 2007) a deficit in comparative studies of lateral offsets that center on the user's experience has been identified (Honig et al., 2018). Furthermore, the majority of existing studies investigate wide following angles with close to human-sized robots (e.g., Morales et al., 2012, 2014; Honig et al., 2016). This approach is reasonable for large robots, which directly communicate with the accompanied person and can be easily seen by other people, but is not as feasible for small mobile robots. Especially in crowded spaces, there is the risk that people overlook small transport robots and e.g., stumble over them.

Therefore, in this study, the interaction between users and a Follow Me robot is researched, with a focus on user experience on a number of subjective variables. To this end, the following distance between the human and the robot is varied two-fold (personal vs. social space) and the lateral offset is varied three-fold (offset to the left vs. center offset vs. offset to the right). While distance and lateral offset (or following angle) have been researched before, the two positional variables have not been investigated for their combined influence on users' experience with small form-factor Follow Me robot in detail. To assess the influence of different experimental conditions on the user, the perceived *comfort, expectancy conformity, human likeness, safety, trust, and unobtrusiveness* is assessed through a questionnaire. Since the Follow Me robot is a highly technical system, and the level of affinity toward technology could influence participants' preferences, the level of *affinity for technology* of participants (Karrer et al., 2009) is surveyed.

## Prototype

**Figure 1:**
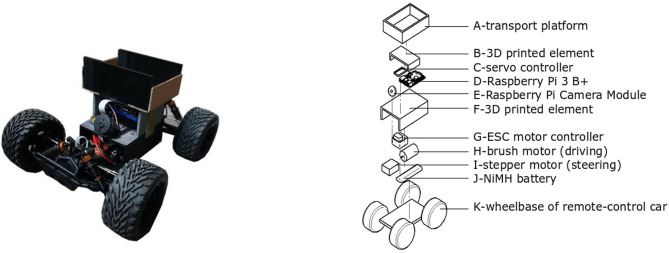
Prototype (Left) and specification of components (Right) of the Follow Me robot.

To test how varying following distances and lateral offsets influence users' perception of a Follow Me robot, a simple prototype robot was built (Figure 1). A remote-control car was used as the basis for the Follow Me robot used in this study. The vehicle is driven by a brush motor, steered by a stepper motor, and powered by a built-in NiMH battery with a capacity of 4,200 mAh. A Raspberry Pi 3 B+, a small single board computer, powered by a separate battery pack, was used to run a script for controlling the robot movement.

Due to the high voltage of the brush and stepper motor, a servo controller (PCA9685 from Adafruit) was used to connect the Raspberry Pi to the brush motor. To control the distance and angle between the robot and the human participant, a Raspberry Pi Camera Module was used to detect the human participant. The camera module had a maximum resolution of 3,280 × 2,464 pixels, which was reduced to 300 × 200 pixels to allow real-time processing of the video data. The camera recorded with 120 color frames per second. An existing package for OpenCV, RaspRobot, was used as the basis for the *follow me* algorithm applied in this study (Oliveira, n.d.; see section Implementation of Tracking). As the experimental design required the robot to carry personal items of participants, a small platform was attached to the top of the robot. All components (Raspberry Pi, battery pack, camera module, and personal item platform) were connected to the wheelbase through custom plastic elements, 3D printed using an *Ultimaker 2*+ printer. A schematic diagram of the components is presented in Figure 1. The robot measured 435 mm (l) × 325 mm (w) × 220 mm (h).

### Implementation of Tracking

To allow accurate visual tracking, without interference by differences in the outside appearance of users of the robot, participants were asked to wear a pink colored reflective vest during the experiment. Using the RaspRobot software package (Oliveira, n.d.) the Raspberry Pi utilized color segmentation in the RGB space to differentiate between the pink color of the reflective vest and the environment. Before the start of the study, the number of pink pixels detected by the camera was related to the distance of the robot users. This pretesting was conducted with robot users of different height, to rule out an influence of outward user appearance on tracking accuracy. Using this approach, pixel thresholds for personal and social distance were identified and written into the OpenCV code. Through adjustment of the robot speed, it was then possible to regulate the robot to human distance through the number of pink pixels detected. While the implemented tracking method allows for a highly accurate detection of the robot to human following distance, the non-linear velocity of human movement together with fluctuating robot acceleration and deceleration does not allow an exact distance following throughout the whole trial. To control for this, following distances were defined as ranges. In the *personal space* experimental condition, the robot was programmed to follow the user with a distance of ~1.2 m, varying by ±15 cm. In the *social space* condition, the robot followed with a distance of ~2.0 m, also varying by ±15 cm.

For the variation of the robot's lateral position in relation to the user, three conditions were programmed. The *left offset* condition resulted in a lateral distance of 50 cm to the left of the user, the *right offset* condition resulted in the same offset to the right of the user, while the *center offset* condition had no lateral offset. Similar to the distance adjustment, the position of the pink pixels in a given video frame was used to control the lateral offset, resulting in a lateral offset in relation to the user.

## Methods

Eighteen participants (50% female) were recruited at the Technische Universität Berlin, resulting in a predominantly student sample with an age ranging from 22 to 30 years (*M* = 24.17; *SD* = 2.15). The majority (*n* = 10) identified their profession as mainly technical and half of the participants were familiar with the concept of Follow Me robots, with one person having already interacted with a Follow Me robot before taking part in the experiment. The study used a 2 (distance) × 3 (lateral offset) design with both factors varied as within-subjects factors, i.e., each participant was presented with all conditions. All 6 conditions were presented in a counterbalanced order to avoid sequence effects.

To investigate how robotic following behavior affected the participant's perception, a questionnaire in German was composed, comprised of items used in previous mobile human-robot interaction research (Lauckner et al., 2014; Honig et al., 2016). Besides a single item measurement of *trust*, the dimensions *perceived comfort, expectation conformity, human likeness, safety*, and *unobtrusiveness* of the robot movements were assessed. A convenience translation of the questionnaire is presented in Table 1. All items were answered on a 7-point Likert scale with poles that expressed complete disagreement and complete agreement (e.g., *1* = *I fully agree; 7* = *I disagree completely*).

**Table 1:** Questionnaire items and dimensions (including Cronbach's α), translated from German.

**Dimension**	**Item**
**Comfort (α** **=0.82)**	
	Did you perceive the distance between the robot and you as unpleasant?
	How did you feel about the distance that the robot chose when approaching you?
	I liked the robot.
	I was satisfied with the way the robot followed me.
	The movement behavior of the robot was good.
	The robot was too slow.
	The speed of the robot was comfortable for me.
	The task was exhausting.
**Expectation conformity (α** **=0.80)**	
	The movement behavior of the robot was predictable.
	The movement behavior of the robot was surprising.
	The robot behaved as I expected.
**Human likeness (α** **=0.46)**	
	How human-like did you perceive the speed of the robot?
	In comparison to a human normally behaving in this situation, the robot was driving:
	The movement of the robot was polite.
**Safety (α** **=0.80)**	
	How safe did you feel in the vicinity of the robot?
	I felt safe with the distance to the robot.
	The movement behavior caused an unpleasant feeling in me.
**Trust**	
	I would trust a robot with comparable distance behavior.
**Unobtrusiveness (α** **=0.79)**	
	I adjusted my speed to the robot.
	I was able to walk undisturbed.
	My walking behavior was not dependent on the robot.
	The robot left me enough free space.
	The robot stressed me.

### Task and Procedure

**Figure 2:**
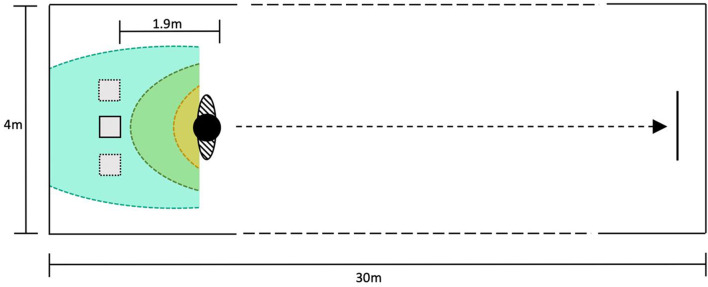
Depiction of the experimental setup with robots and humans start positions, as well as the interpersonal distances in human-human interaction for northern Europeans (Hall, 1966); yellow-intimate (0–45 cm); green-personal (45–120 cm); cyan-social (120–360 cm) (robot always started in the social zone).

The aim of the human-robot collaboration was to transport a personal item (e.g., mobile phone or wallet) belonging to the participant through a hallway. First, after filling out a short sociodemographic questionnaire, participants familiarized themselves with the robot, by observing the robot moving around. Afterwards, the participants were equipped with the high-visibility vest and instructed to place a valuable personal item on the transport box of the robot. Two examples for a valuable item were listed, a mobile phone or wallet, and all participants decided to use their mobile phone for the task. Every trial started with the robot stationary behind the participant in a fixed distance, but with the condition specific lateral offset. As soon as the participants started moving in walking pace from the initial position the individual trial was initialized. Participants walked for ~27 m and stopped once they had reached a colored mark on the ground (Figure 2). The six different robot movement combinations (distance and angle) were presented in a counterbalanced order. After each trial, participants filled out a questionnaire (Table 1) about their subjective experience regarding the robot's movement behavior on a laptop. Finally, after the completion of all conditions, participants were asked to indicate their preferred following condition, and filled out a questionnaire on their affinity for technology (Karrer et al., 2009).

### Analysis

All rating data except the single item trust dimension were analyzed by a two-way (2 × 3) repeated-measures analysis of variance (ANOVA), with distance (within-subjects; personal vs. social space) and lateral offset (within-subjects; left, center, right) as independent variables and perceived comfort, expectancy conformity, human likeness, safety, and unobtrusiveness as dependent variables. If Mauchly's test indicated that the assumption of sphericity had been violated, degrees of freedom were corrected using Greenhouse–Geisser estimates of sphericity. The influence of the following distance on the trust dimension was analyzed using a Wilcoxon test, and the influence of the lateral offset on trust was analyzed using a Friedman test.

## Results

Mean ratings and standard deviation of all dependent variables for all experimental conditions are presented in Table 2. We used Cronbach's α to assess the internal consistency of the proposed item groups in our questionnaire and received acceptable to good consistencies for comfort, expectation conformity, safety and unobtrusiveness (α = 0.79–0.82). For the human likeness scale Cronbach's α was unacceptable (α = 0.46), mainly due to the item on the politeness of the robot's movement. Without this item the internal consistency would have been acceptable (α = 0.72). To assess whether there was an effect of sequence despite the counterbalanced experimental conditions, we calculated an ANOVA for all parametric dependent variables (averaged for dimensions) and a Friedman test for the trust variable, with the chronological sequence of conditions as the independent variable. We found no significant influence of chronological order of conditions on the dependent variables (all *p* > 0.23). All data is available in the repository of the Open Science Framework (Siebert et al., 2020).

**Table 2:** Mean subjective ratings of all experimental conditions, parentheses show standard deviation.

	**Personal**			**Social**		
**Variable**	**Left**	**Center**	**Right**	**Left**	**Center**	**Right**
Comfort	5.09 (1.00)	5.44 (0.97)	5.17 (1.07)	5.51 (0.87)	5.56 (0.69)	5.4 (0.65)
Expectation conformity	3.89 (1.25)	4.89 (1.23)	4.56 (1.07)	4.63 (1.26)	5.06 (1.04)	4.39 (0.95)
Human likeness	4.67 (0.91)	4.74 (1.02)	4.7 (0.87)	4.98 (0.7)	5.07 (0.9)	5.04 (0.76)
Safety	5.08 (0.88)	5.30 (1.23)	5.28 (1.13)	5.48 (0.85)	5.52 (1.19)	5.63 (0.80)
Trust	4.89 (1.23)	5.06 (1.21)	5.11 (1.49)	5.39 (1.15)	5.44 (1.19)	5.28 (1.13)
Unobtrusiveness	5.07 (1.02)	4.99 (1.3)	4.97 (1.27)	5.1 (0.87)	5.56 (1.17)	5.42 (0.97)

### Distance

The analyses revealed significant main effects of robots spatial distance for comfort [*F*_(1,17)_ = 7.74; *p* = 0.044; $\eta_{\text{p}}^{\text{2}}$ = 0.218], human likeness [*F*_(1,17)_ = 6.24; *p* = 0.023; $\eta_{\text{p}}^{\text{2}}$ = 0.269], trust (*Z* = −2.3; *p* = 0.022), and unobtrusiveness [*F*_(1,17)_ = 6.39; *p* = 0.022; $\eta_{\text{p}}^{\text{2}}$ = 0.273). For all these variables, the social following distance was rated higher and therefore more positive than the personal following distance. Descriptively, this effect can also be found for the variables of perceived safety, for which the main effect of human-robot distance just failed to reach significance [*F*_(1,17)_ = 4.47; *p* = 0.05; $\eta_{\text{p}}^{\text{2}}$ = 0.208].

### Lateral Offset

The analyses revealed a significant main effect of lateral offset only for expectancy conformity [*F*_(1.37,23.2)_ = 5.65; *p* = 0.018; $\eta_{\text{p}}^{\text{2}}$ = 0.249]. The expectation conformity was significantly higher in the centered condition (*M* = 4.95; *SE* = 0.247), compared to the left (*M* = 4.26; *SE* = 0.257), and right (*M* = 4.47; *SE* = 0.177) lateral conditions (both *p* < 0.001 after Bonferroni correction for multiple comparisons).

### Affinity for Technology

Affinity for technology was measured as a control variable and showed no significant correlation with any of the dependent variables. However, there was a correlation of affinity to technology and *post-hoc* indicated preferred distance (*r* = −0.575; *p* < 0.05). To explore this correlation further, a Chi-square test was conducted and revealed significant association between affinity to technology and preferred distance [$\chi_{\left(1\right)}^{2}$ = 5.95, *p* = 0.015]. Most Participants with high affinity of technology preferred personal distance (70%), whereas most participants with low affinity of technology (87.5%) preferred social distance to the robot.

## Discussion

In this study, we investigated the influence of distance and lateral offset in the interaction between humans and Follow Me robots. The analysis of the subjective variables collected from 18 participants revealed an overall preference for Follow Me robots to follow in the *social space*, i.e., with a relatively large distance of ~2 m. This significant preference for a larger following distance for participants' ratings of *comfort, human likeness, trust*, and *unobtrusiveness* was not expected. Since the Follow Me robot in this study carried a valuable personal item of the user, it was hypothesized that a closer following distance would be preferred. A potential cause for this preference for a larger following space in comparison to human-human interaction could be uncertainty in the ability of the Follow Me robot to stop in time. While humans are naturally capable to adjust their walking speed to avoid running into another human, humans might not have the same confidence in Follow Me robots. Our results on affinity for technology support this hypothesis, as participants with a high affinity for technology prefer following distances in the personal space to a higher degree than participants with a low affinity for technology. As this individual tendency characterizes the engagement or avoidance of human-technology interaction, participants with higher affinity might have a higher system knowledge and familiarity with similar robotic devices, whereas participants with low affinity for technology might be more skeptical toward the abilities of the robot. A similar effect has been found in earlier studies, where users' negative attitudes and anxiety toward robots increased users' preferred distances to robots (Nomura et al., 2007). Our finding on the influence of affinity for technology on preferred distances underlines the relevance of prior exposure in spatial human-robot interaction, as people tend to adapt their spatial behavior and comfort perception in the first interactions and then stabilize their interaction behavior (Walters et al., 2011).

For the lateral offset of the robot, the center position in which the Follow Me robot follows the participant without any offset was preferred by users regarding the expectation conformity. I.e., robot following with a lateral offset of 50 cm to the left or the right of participants significantly decreased participants' ratings on movement expectation of robot movement. This result has to be seen in the light of the three items incorporated in the dimension of expectation conformity (predictability of the robot; a lack of surprises in movements; compliance with expected behavior; Table 1). These mainly indicate a comparison of the robot's behavior with existing expectations toward robots. As such, ratings will be heavily influenced by prior exposure to robots and derived expectations for lateral offsets. Hence, the results on lateral offset and expectation conformity present a retrospective view on robots' movement behavior and do not necessarily present an indication of generally acceptable robot movement conventions. As lateral offset in this study had no significant influence on other subjective variables, it appears that lateral offset deviations in the range of ± 50 cm in relation to the user are not critical to users' acceptance of the robot.

While the variation of distance and lateral offset influenced participants' ratings of the robot, the overall ratings for all experimental conditions were relatively high (Table 2). This might be an indication that the distance and lateral offset variation in this study represents a range of robot movement that is generally perceived with a positive valence by users. While the choice of following distance and lateral offset was theory driven, the experimental environment restricted the range in which distance and offset could be varied. Future studies should incorporate movement variables of a broader range.

There are a number of limitations to this study. This study exclusively uses self-reported measures for rating the human-robot interaction. Behavioral measures, such as tracking of the walking speed of users or eye-gaze toward the robot would provide a more comprehensive mixed methods picture of the human-robot interaction. The environment in this study was rather simple, consisting of a long hallway without other foot-traffic. In real-world applications of Follow Me robots, environments will be crowded, which could potentially influence users' preferences for following distances and lateral offset. On the hardware side, the robots tracking mechanism needs to be changed to allow tracking of users in real-live environments, and tracking accuracy should be assessed within the experiment. The nature of this study is incremental and adds to the existing literature on spatial human-robot interaction by varying two variables in an experimental setting with a comparatively small Follow Me robot. At the same time, the lack of open ended questions in the follow-up questionnaire limits the identification of influencing variables on users' experience to the pre-selected closed questions. Future studies should include open ended questions to be able to capture other variables that might influence users' perception of the interaction with the Follow Me robot.

In conclusion, the results of this study emphasize the importance of individual users' subjective attitudes toward technology in human-robot interaction. Future research needs to take the heterogeneity of the users into account by applying adaptable or adaptive robotic following behavior. Hence, the acceptance and broad usage of Follow Me robots hinges on a flexible following behavior depending on interaction context, the interaction goal, and the preference of an individual user.

## Author Contributions

All authors listed have made a substantial, direct and intellectual contribution to the work, and approved it for publication.

## Conflict of Interest

The authors declare that the research was conducted in the absence of any commercial or financial relationships that could be construed as a potential conflict of interest.
